# Topiramate Reprofiling for the Attenuation of Cadmium-Induced Testicular Impairment in Rats: Role of NLRP3 Inflammasome and AMPK/mTOR-Linked Autophagy

**DOI:** 10.3390/ph15111402

**Published:** 2022-11-14

**Authors:** Hany H. Arab, Hayat A. Abd El Aal, Shuruq E. Alsufyani, Azza A. K. El-Sheikh, El-Shaimaa A. Arafa, Ahmed M. Ashour, Ahmed M. Kabel, Ahmed H. Eid

**Affiliations:** 1 Department of Pharmacology and Toxicology, College of Pharmacy, Taif University, P.O. Box 11099, Taif 21944, Saudi Arabia; 2 Department of Pharmacology, Egyptian Drug Authority (EDA), Formerly NODCAR, Giza 12654, Egypt; 3 Basic Health Sciences Department, College of Medicine, Princess Nourah bint Abdulrahman University, P.O. Box 84428, Riyadh 11671, Saudi Arabia; 4 College of Pharmacy and Health Sciences, Ajman University, Ajman 346, United Arab Emirates; 5 Center of Medical and Bio-Allied Health Sciences Research, Ajman University, Ajman 346, United Arab Emirates; 6 Department of Pharmacology and Toxicology, College of Pharmacy, Umm Al Qura University, P.O. Box 13578, Makkah 21955, Saudi Arabia; 7 Department of Pharmacology, Faculty of Medicine, Tanta University, Tanta 31527, Egypt

**Keywords:** topiramate, cadmium, testicular damage, NLRP3 inflammasome, apoptosis: autophagy

## Abstract

Topiramate, a promising drug classically used for the management of neurological disorders including epilepsy and migraine, has demonstrated marked anti-inflammatory and anti-apoptotic actions in murine models of cardiac post-infarction inflammation, wound healing, and gastric/intestinal injury. However, its potential impact on cadmium-induced testicular injury remains to be elucidated. Herein, the present study aimed to explore the effect of topiramate against cadmium-invoked testicular impairment with emphasis on the molecular mechanisms linked to inflammation, apoptosis, and autophagy. Herein, administration of topiramate (50 mg/kg/day, by gavage) continued for 60 days and the testes were examined by histology, immunohistochemistry, and biochemical assays. The present data demonstrated that serum testosterone, sperm count/abnormalities, relative testicular weight, and histopathological aberrations were improved by topiramate administration to cadmium-intoxicated rats. The rescue of testicular dysfunction was driven by multi-pronged mechanisms including suppression of NLRP3/caspase-1/IL-1β cascade, which was evidenced by dampened caspase-1 activity, lowered IL-1β/IL-18 production, and decreased nuclear levels of activated NF-κBp65. Moreover, curbing testicular apoptosis was seen by lowered Bax expression, decreased caspase-3 activity, and upregulation of Bcl-2. In tandem, testicular autophagy was activated as seen by diminished p62 SQSTM1 accumulation alongside Beclin-1 upregulation. Autophagy activation was associated with AMPK/mTOR pathway stimulation demonstrated by decreased mTOR (Ser2448) phosphorylation and increased AMPK (Ser487) phosphorylation. In conclusion, combating inflammation/apoptosis and enhancing autophagic events by topiramate were engaged in ameliorating cadmium-induced testicular impairment.

## 1. Introduction

Cadmium (Cd) is a non-essential heavy metal whose exposure is unavoidable in humans due to its extensive occupational and non-occupational sources [1]. Despite the fact that cadmium toxicity affects many organs such as lungs, liver, and kidneys, testes are particularly sensitive [2,3]. Cadmium triggers negative effects on the reproductive organs, resulting in infertility [1,4]. The integrity of the blood–testes barrier has previously been documented to be substantially compromised by cadmium exposure [5]. In this context, cadmium poisoning has been linked to impaired testicular functioning, particularly defective production of male androgens [4]. Previous research has also shown that cadmium treatment reduces the quality and quantity of sperm in animals [2,5]. Notably, seminal cadmium levels have been correlated with the oligo-zoospermia seen in idiopathic male infertility [1,4,5]. Additionally, cadmium instigates several testicular abnormalities, including testicular steroidogenesis abrogation, Sertoli cell dysfunction, germ cell death, and ultimately, prostate cancer [1,4,5].

As part of the inflammatory response and the innate immune system, inflammasomes play a key role in the pathophysiology and progression of testicular injury. The most prevalent type of inflammasome is the nucleotide-binding domain (NOD)-like receptor family, pyrin domain-containing 3 (NLRP3) inflammasome [6]. The NLRP3 inflammasome is a multiprotein complex made up of three protein subunits: a sensor molecule called NLRP3, an adapter protein called ASC, and an effector protein called caspase 1. In the context of testicular pathologies, the crosstalk between NLRP3 inflammasome and autophagy has been established. To put this into perspective, autophagy stimulation has been reported to curtail NLRP3 inflammasome activity and associated pro-inflammatory responses [7]. 

Excessive testicular pro-inflammatory events culminate in provoking the mitochondrial apoptotic cell death in germ cells, Leydig cells, and Sertoli cells [7,8,9]. Indeed, apoptosis has been linked to the pathogenesis of cadmium-induced testicular disruption where pro-apoptotic processes prevail. In this regard, overexpression of Bcl-2-associated x protein (Bax) and the downregulation of the anti-apoptotic B cell lymphoma-2 protein (Bcl-2) have been described [3,10]. 

Several reports demonstrate that autophagy is a stress-adaptation process that prevents cell death and abrogates apoptosis. Conversely, other studies describe autophagy as a cell-death route and that autophagic breakdown is a newly characterized mechanism of cell death [11,12,13]. Indeed, autophagy is seen as a double-edged sword since it has diverse functions under different physiological or pathological conditions. In the context of testicular disorders, both hyperactive autophagy [14] and impaired autophagy [13,15] have been disclosed in rodent models of testicular injury. In the context of cadmium-evoked testicular toxicity, curtailed autophagy flux has been observed despite the overexpression of numerous autophagy-linked markers, including Beclin 1 [13,15,16]. To put this into perspective, cadmium has been demonstrated to impair autophagy by disrupting calcium-dependent autophagosome-lysosome fusion [17] and by augmenting lysosomal membrane permeability [18]. For tissue homeostasis, autophagy and apoptosis have been demonstrated to be tightly controlled [14,19]. Apoptosis is regarded to be a death-promoting mechanism for eliminating severely damaged cells, whereas autophagy increases the survival potential of cells in response to several stressors [11,19]. The crosstalk between autophagy and apoptosis has been demonstrated in several testicular disorders [13,20]. In testicular dysfunction, autophagy has been demonstrated to dampen apoptotic cell death [21].

**Figure 1:**
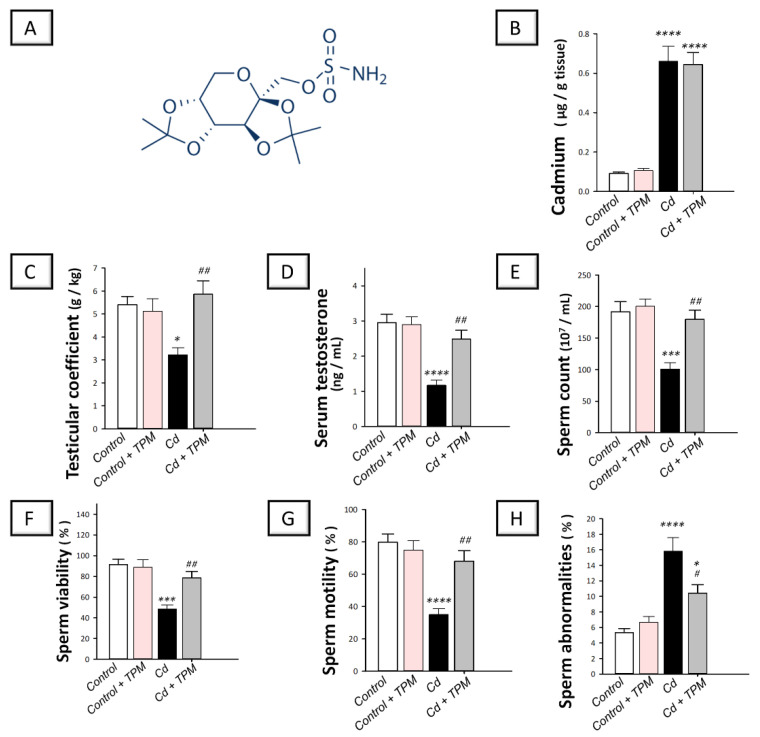
Effect of cadmium and/or topiramate on testicular impairment and sperm analysis in rats. The co-treatment with topiramate (chemical structure; **A**) did not significantly affect cadmium testicular levels [H (3, 20) = 42.45, *p* < 0.0001] (**B**). However, topiramate cotreatment counteracted the testicular impairment and sperm aberrations that were activated by cadmium administration. This was revealed by increased testicular coefficient [H (3, 20) = 6.23, *p* < 0.0001] (**C**), serum testosterone [H (3, 20) = 14.32, *p* < 0.0001] (**D**), sperm count [H (3, 20) = 12.20, *p* < 0.0001] (**E**), sperm viability [H (3, 20) = 11.26, *p* = 0.0002] (**F**), and sperm motility [H (3, 20) = 13.93, *p* < 0.0001] (**G**). Meanwhile, the sperm abnormalities were lowered [H (3, 20) = 17.67, *p* < 0.0001] (**H**). Results are demonstrated as the mean ± standard error of the mean (SEM) for six rats in each experimental group. * *p* < 0.05, *** *p* < 0.001, or **** *p* < 0.0001 signifies statistical significance versus the control group; ^#^ *p* < 0.05, ^##^ *p* < 0.01, signifies statistical significance versus cadmium chloride-treated group. TPM, topiramate; Cd, cadmium chloride.

Topiramate, a sulfamate-substituted monosaccharide drug (Figure 1A depicts its chemical structure), has been originally approved for the treatment of epilepsy. Moreover, it has shown efficacy against several neurological disorders, including pain syndromes and migraine prophylaxis. Mechanistically, topiramate has demonstrated several neuroprotective mechanisms, including augmentation of γ-aminobutyric acid (GABA) receptor activity, inhibition of α-amino-3-hydroxy-5-methyl-4-isoxazole-propionate (AMPA) subtype of the excitatory glutamate receptors, blockade of voltage-gated sodium channels, and inhibition of L-type voltage-gated calcium ion channels [22,23]. Growing evidence revealed that topiramate is endowed with marked anti-inflammatory [22,24,25,26], and anti-apoptotic features [27,28,29] that mediated its efficacy against diverse pathologies in the experimental setting. Beyond its reported beneficial actions in the nervous system, topiramate’s anti-inflammatory and anti-apoptotic actions have intercepted its efficacy for the mitigation of renal damage in apo-E-deficient mice [22], post-infarction ventricular remodeling/cardiac rupture [24], experimental abdominal aortic aneurysm [25], and wound healing [26]. Despite the promising effects of topiramate in the pre-clinical setting, no previous work has addressed its potential role in cadmium-induced testicular impairment. In particular, the molecular mechanisms related to the effect of topiramate on autophagy are inadequately examined. Thus, the current study aimed at examining the potential of topiramate to attenuate cadmium-induced testicular dysfunction. Herein, the impaired testicular function and sperm parameters were studied. At the cellular and molecular levels, the mechanisms pertaining to testicular NLRP3-driven inflammation, apoptosis, and AMPK/mTOR-associated autophagy were explored.

## 2. Results

### 2.1. Effect of Topiramate on Testicular Dysfunction and Impaired Spermatogenesis in Cadmium-Induced Testicular Injury in Rats

In cadmium-intoxicated rats, the testicular level of cadmium metal was significantly increased by 623.4% (*p* < 0.0001) as illustrated in Figure 1B. More importantly, cadmium intoxication triggered testicular impairment where the testicular coefficient and serum testosterone levels were significantly lowered by 40.2% (*p* < 0.05) and 60.3% (*p* < 0.0001), respectively, compared to control rats, as described in Figure 1C,D. In the same direction, Figure 1E–H, characterizes that the sperm count, viability, and motility were significantly diminished by 47.4% (*p* < 0.001), 46.9% (*p* < 0.001), and 56.1% (*p* < 0.0001), respectively, whereas the sperm abnormalities were significantly increased in cadmium-intoxicated rats by 196.9% (*p* < 0.0001). Except for testicular cadmium levels, topiramate administration reversed these changes. These data reveal that the testicular dysfunction and impaired spermatogenesis in cadmium-intoxicated rats were relieved by topiramate treatment.

### 2.2. Effect of Topiramate on the Histopathological Aberrations in Cadmium-Induced Testicular Injury in Rats

**Figure 2:**
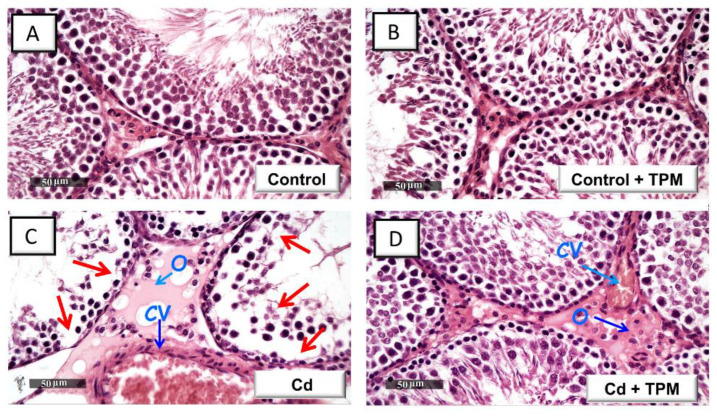
Topiramate ameliorates the histologic aberrations in cadmium-induced testicular injury in rats. The testicular histomorphological changes were examined by microscopy after staining with hematoxylin and eosin (H-E) stain. Typical morphology of the seminiferous tubules, their germinal epithelium lining, and interstitial tissue were observed in the control group (**A**) and topiramate-treated control group (**B**). In cadmium-intoxicated rats, severe atrophy of the seminiferous tubules, notable degeneration of the germinal cells (red arrow), interstitial edema (O), and congestion of interstitial blood vessels (CV) were observed (**C**). These histologic changes were mitigated by administration of topiramate as manifested by the typical seminiferous tubules which were lined with intact germinal epithelium (**D**). However, focal areas of mild interstitial edema (O) and congested blood vessels (CV) were still observed. TPM, topiramate; Cd, cadmium chloride.

The histologic images in the control and topiramate-treated control groups revealed that the seminiferous tubules were active with intact and well-organized germinal epithelium manifesting various stages of maturation/development. Moreover, the basement membrane, vasculature, and interstitial tissues were intact (Figure 2A,B). In cadmium-intoxicated rats, disorganization and atrophy of the seminiferous tubules, diffuse germinal cell degeneration, germ cell vacuolization, nuclear pyknosis, loss of mature spermatids, interstitial edema, and congestion of interstitial blood vessels were detected (Figure 1C). These histologic changes were attenuated by the administration of topiramate, as manifested by the typical seminiferous tubules which were lined with intact germinal epithelium exhibiting various stages of maturation/development (Figure 2D). However, mild persistence of interstitial edema and some congested blood vessels were still observed in discrete areas of the testicular tissue in topiramate-treated testicular injury group. 

### 2.3. Effect of Topiramate on Testicular NLRP3 Inflammasome Activation in Cadmium-Induced Testicular Injury in Rats

**Figure 3:**
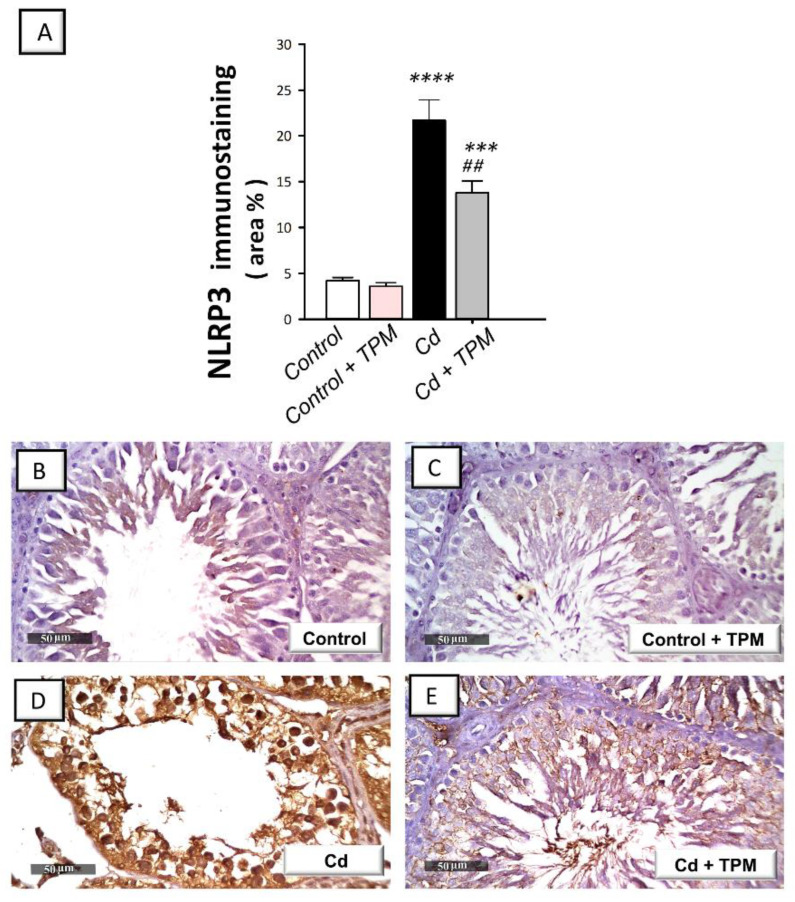
Effect of cadmium and/or topiramate on NLRP3 protein expression in testes of rats. The co-treatment with topiramate lowered the testicular protein expression of NLRP3 which was increased by cadmium administration. The determination of NLRP3 protein expression was carried out by detection of its immunostaining (brown color) in testes of animals at 400× magnification. The slides were counterstained with hematoxylin. (**A**) Quantitative analysis of NLRP3 immunostaining where topiramate lowered the immunostaining of NLRP3 [H (3, 20) = 43.31, *p* < 0.0001]. The quantification was presented as the area % of the brown immunostaining relative to the microscopic field’s whole area (in each section, six non-overlapping fields were investigated). Results are demonstrated as the mean ± standard error of the mean (SEM). *** *p* < 0.001, or **** *p* < 0.0001 signifies statistical significance versus the control group; ^##^ *p* < 0.01 signifies statistical significance versus cadmium chloride-treated group. (**B**,**C**) Representative photomicrographs showing minimal expression in the control and topiramate-treated control groups, respectively. (**D**) Representative photomicrographs showing increased immunostaining of NLRP3 in cadmium-intoxicated group. (**E**) Representative photomicrographs showing that topiramate downregulated the protein expression of NLRP3 as indicated by lowered immunostaining. NLRP3, the nucleotide-binding domain (NOD)-like receptor family, pyrin domain-containing 3. TPM, topiramate; Cd, cadmium chloride.

**Figure 4:**
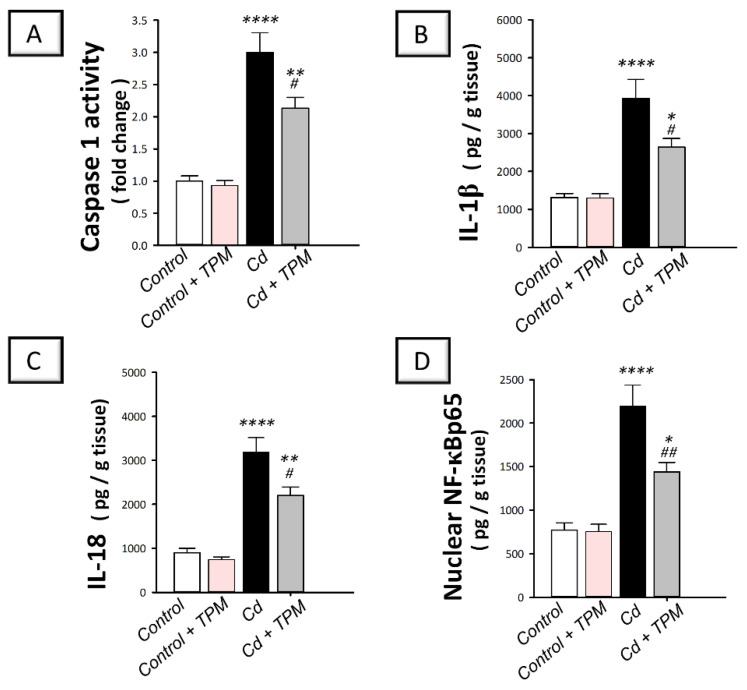
Effect of cadmium and/or topiramate on caspase-1/IL-1β/NF-κB pathway in testes of rats. The co-treatment with topiramate suppressed testicular caspase-1/IL-1β pathway that was activated by cadmium administration. This was revealed by lowered activity of caspase 1 [H (3, 20) = 29.61, *p* < 0.0001] (**A**) and decreased levels of the pro-inflammatory IL-1β [H (3, 20) = 18.93, *p* < 0.0001] (**B**), and IL-18 [H (3, 20) = 31.92, *p* < 0.0001] (**C**). Meanwhile, the co-treatment with topiramate inhibited NF-κB activation and lowered the nuclear levels of pro-inflammatory signal NF-κB p65 [H (3, 20) = 22.35, *p* < 0.0001] (**D**). Results are demonstrated as the mean ± standard error of the mean (SEM) for six rats in each experimental group. * *p* < 0.05, ** *p* < 0.01, **** *p* < 0.0001 signifies statistical significance versus the control group; ^#^ *p* < 0.05, ^##^ *p* < 0.01 signifies statistical significance versus cadmium chloride-treated group. IL-1β, interleukin-1 beta; IL-18, interleukin 18; NF-κB, nuclear factor kappa B. TPM, topiramate; Cd, cadmium chloride.

In cadmium-intoxicated rats, the testicular pro-inflammatory NLRP3/caspase-1/IL-1β pathway was activated compared to control rats. This was seen by a significant increase in NLRP3 immunostaining (by 417.4%, *p* < 0.0001, Figure 3A), caspase-1 activity (by 200.4%, *p* < 0.0001), and the pro-inflammatory levels of IL-1β (by 199%, *p* < 0.0001), and IL-18 (by 255.1%, *p* < 0.0001) as demonstrated in Figure 4A–C. Moreover, NF-κB pathway was stimulated as seen by a significant increase in the nuclear levels of activated NF-κB p65 pro-inflammatory signal (by 184.4%, *p* < 0.0001, Figure 4D). These changes were attenuated, and NLRP3/ASC/caspase-1 pathway was suppressed by topiramate administration, as demonstrated by a significant decrease in NLRP3 immunostaining (by 36.3%, *p* < 0.01), caspase-1 activity (by 29%, *p* < 0.05), the pro-inflammatory levels of IL-1β (by 32.9%, *p* < 0.05) and IL-18 (by 30.9%, *p* < 0.05), and the nuclear levels of NF-κB p65 (by 34.4%, *p* < 0.01). These findings indicate that the suppression of NLRP3 inflammasome by topiramate is, at least partly, involved in the mitigation of cadmium-induced testicular injury in rats.

### 2.4. Effect of Topiramate on Testicular Apoptosis Markers in Cadmium-Induced Testicular Injury in Rats

**Figure 5:**
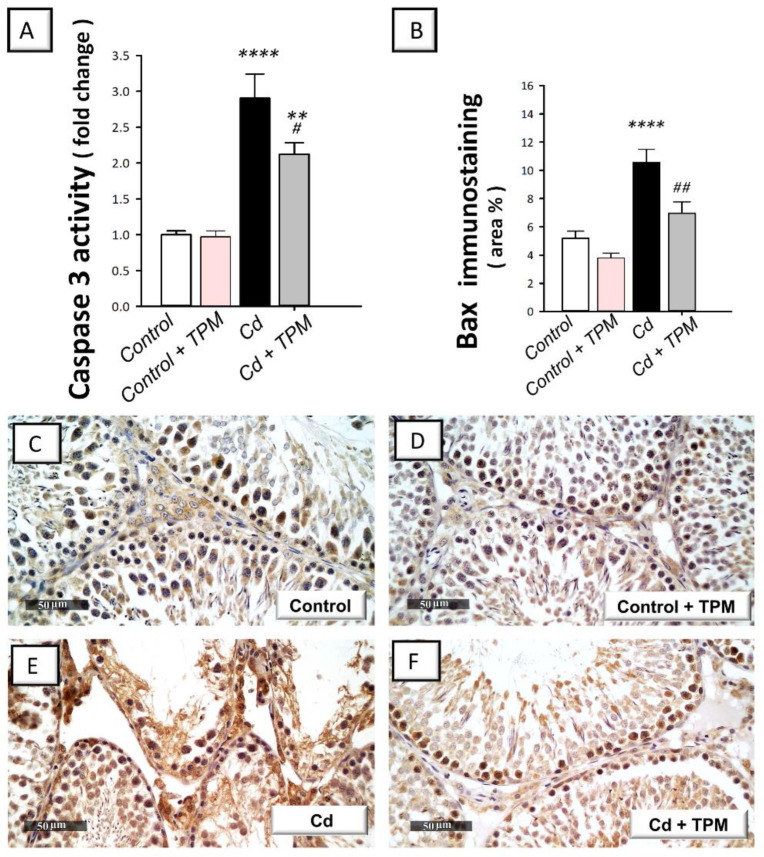
Effect of cadmium and/or topiramate on the pro-apoptotic caspase-3 activity and Bax immunostaining in testes of rats. The co-treatment with topiramate lowered the testicular activity of caspase-3 [H (3, 20) = 23.52, *p* < 0.0001] (**A**) and the quantification of Bax protein expression [H (3, 20) = 18.54, *p* < 0.0001] (**B**) that were increased by cadmium administration. The determination of Bax protein expression was carried out by the detection of its immunostaining (brown color) in the testes of animals at 400× magnification. The slides were counterstained with hematoxylin. (**B**) Quantitative analysis of Bax immunostaining. The quantification was presented as the area % of the brown immunostaining relative to the microscopic field’s whole area (in each section, six non-overlapping fields were investigated). Results are demonstrated as the mean ± standard error of the mean (SEM). ** *p* < 0.01, **** *p* < 0.0001 signifies statistical significance versus the control group; ^#^ *p* < 0.05, ^##^ *p* < 0.01 signifies statistical significance versus cadmium chloride-treated group. (**C**,**D**) Representative photomicrographs showing minimal expression in the control and topiramate-treated control groups, respectively. (**E**) Representative photomicrographs showing increased immunostaining of Bax in cadmium-intoxicated group. (**F**) Representative photomicrographs showing that topiramate downregulated the protein expression of Bax as indicated by lowered immunostaining. Bax. Bcl-2-associated x- protein. TPM, topiramate; Cd, cadmium chloride.

**Figure 6:**
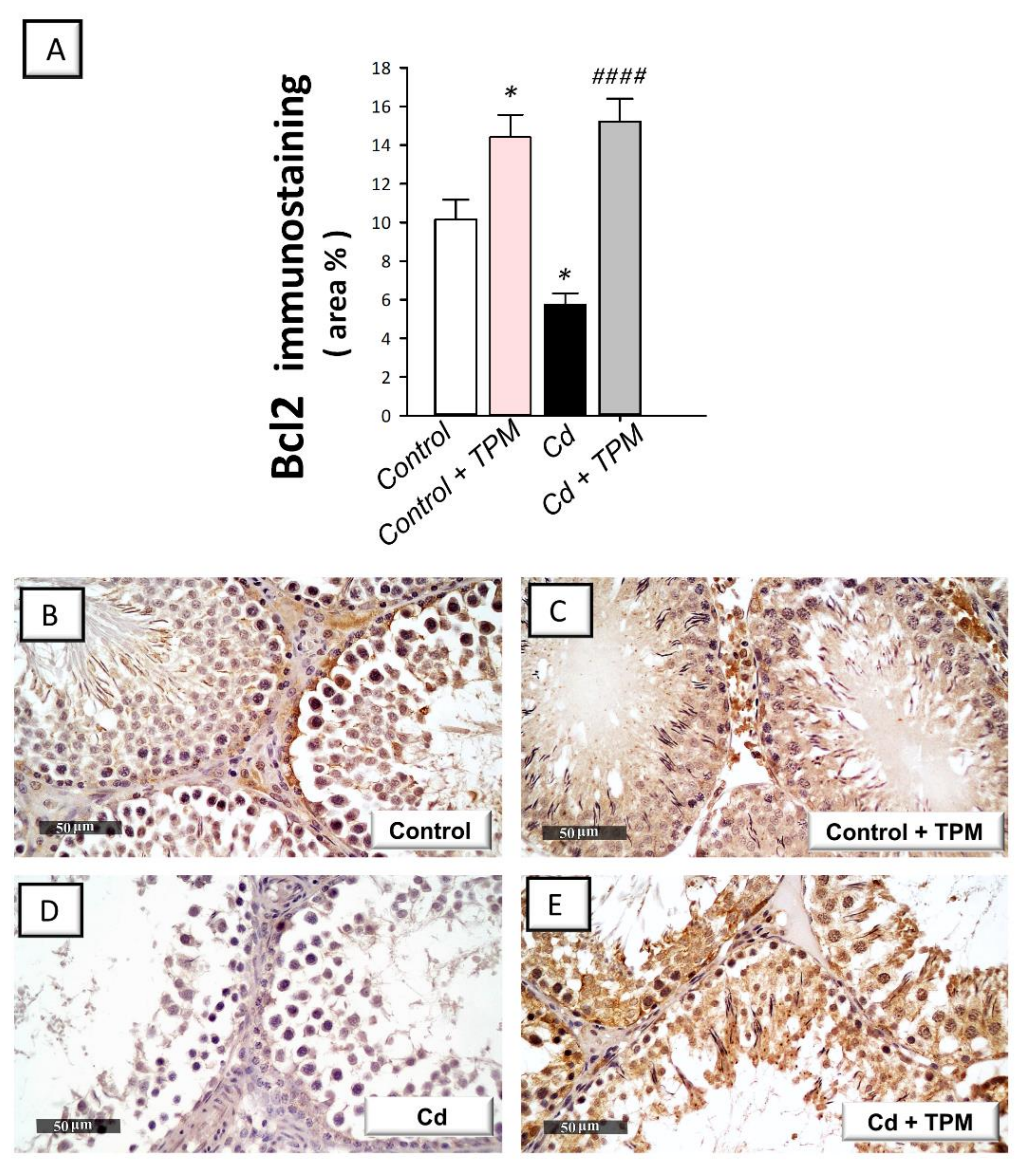
Effect of cadmium and/or topiramate on the immunostaining of the anti-apoptotic Bcl-2 in testes of rats. The co-treatment with topiramate enhances the quantification of testicular Bcl-2 protein expression [H (3, 20) = 18.25, *p* < 0.0001] (**A**) that were lowered by cadmium administration. The determination of Bcl-2 protein expression was carried out by the detection of its immunostaining (brown color) in the testes of animals at 400× magnification. The slides were counterstained with hematoxylin. (**A**) Quantitative analysis of Bcl-2 immunostaining. This was demonstrated as the area % of the brown immunostaining relative to the microscopic field’s whole area (in each section, six non-overlapping fields were investigated). Results are demonstrated as the mean ± standard error of the mean (SEM). * *p* < 0.05 signifies statistical significance versus the control group; ^####^ *p* < 0.001 signifies statistical significance versus the cadmium chloride-treated group. (**B**,**C**) Intense expression in the control and topiramate-treated control groups, respectively. (**D**) Suppressed immunostaining of Bcl-2 in cadmium-intoxicated group. (**E**) Topiramate upregulated the protein expression of Bcl-2 as indicated by increased immunostaining. Bcl-2, B cell lymphoma-2. TPM, topiramate; Cd, cadmium chloride.

In cadmium-intoxicated rats, the testicular pro-apoptotic markers were increased compared to control rats. This was revealed by a significant increase in caspase-3 activity (by 190.6%, *p* < 0.0001) and Bax immunostaining (by 104.2%, *p* < 0.0001, Figure 5A,B) together with a significant decline in Bcl-2 immunostaining (by 43.4%, *p* < 0.05, Figure 6A). These pro-apoptotic alterations were reversed by topiramate administration as seen by a significant decrease in caspase-3 activity (by 27%, *p* < 0.05) and Bax immunostaining (by 34.3%, *p* < 0.01) together with a significant increase in Bcl-2 immunostaining (by 164.4%, *p* < 0.0001). These data reveal that dampening of testicular pro-apoptosis signals by topiramate is, at least partly, involved in the amelioration of cadmium-induced testicular injury in rats.

### 2.5. Effect of Topiramate on Defective Autophagy in Cadmium-Induced Testicular Injury in Rats

**Figure 7:**
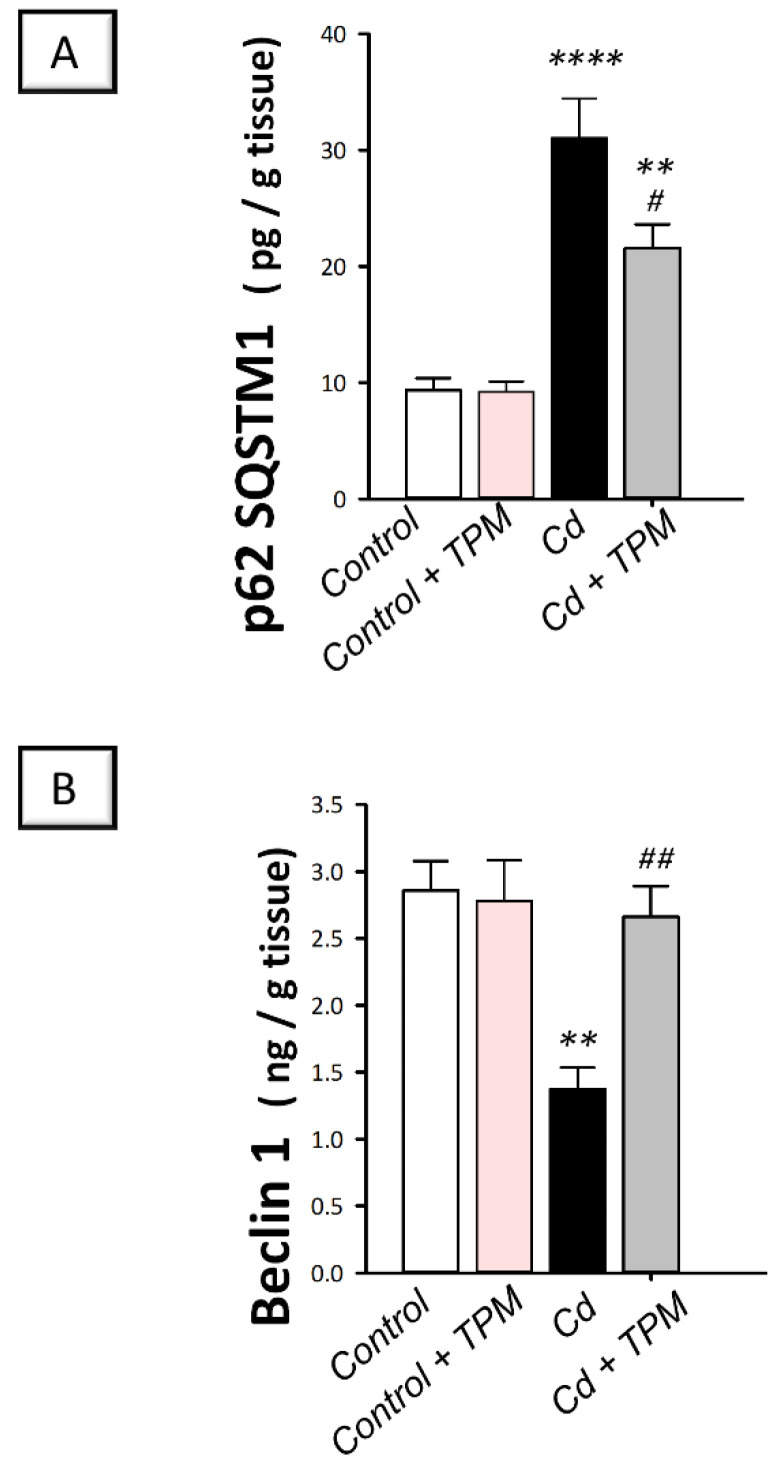
Effect of cadmium and/or topiramate on autophagy markers in testes of rats. The co-treatment with topiramate activated testicular autophagy that was inhibited by cadmium administration. This was manifested by the lowered accumulation of the negative autophagy marker p62 SQSTM1 [H (3, 20) = 25.42, *p* < 0.0001] (**A**) and upregulated protein expression of the positive autophagy marker Beclin 1 [H (3, 20) = 8.80, *p* = 0.0006] (**B**). Results are demonstrated as the mean ± standard error of the mean (SEM) for six rats in each experimental group. ** *p* < 0.01, **** *p* < 0.0001 signifies statistical significance versus the control group; ^#^ *p* < 0.05, ^##^ *p* < 0.01 signifies statistical significance versus cadmium chloride-treated group. p62 SQSTM1, sequestome 1 protein 62. TPM, topiramate; Cd, cadmium chloride.

In cadmium-intoxicated rats, cadmium intoxication instigated a significant accumulation of the negative autophagy marker p62 SQSTM1 (by 232.5%, *p* < 0.0001, Figure 7A) together with a significant decline in Beclin 1 protein expression (by 51.9%, *p* < 0.01, Figure 7B), compared to control rats. These aberrations were reversed, and autophagy flux was enhanced by topiramate administration, as seen by a significant decrease in p62 SQSTM1 protein expression (by 30.5%, *p* < 0.05) together with a significant increase in Beclin 1 protein expression (by 93.6%, *p* < 0.01). These findings reveal that the enhancement of testicular pro-autophagy Beclin 1 and decline of the negative autophagy signal p62 SQSTM1 by topiramate are, at least partly, involved in the amelioration of cadmium-induced testicular injury in rats.

### 2.6. Effect of Topiramate on AMPK/mTOR Pathway in Cadmium-Induced Testicular Damage in Rats

**Figure 8:**
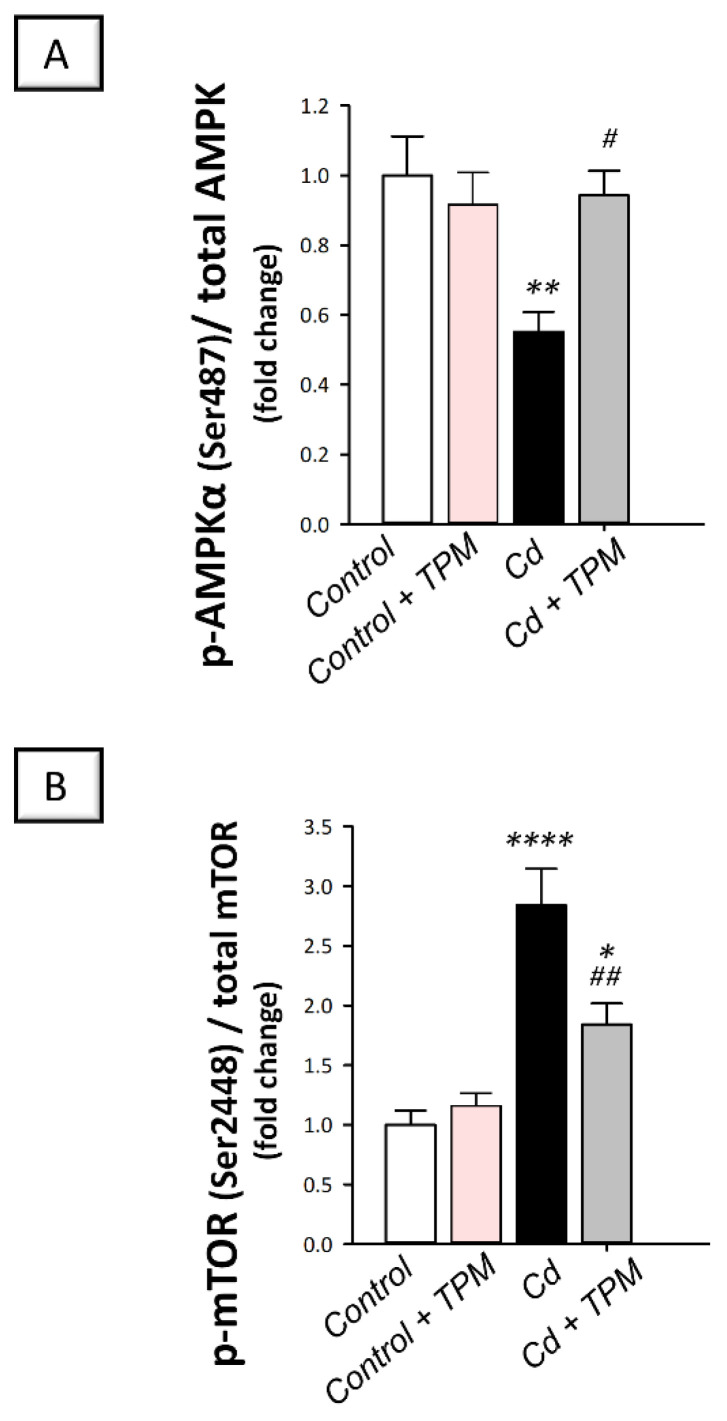
Effect of cadmium and/or topiramate on AMPK/mTOR pathway in testes of rats. The co-treatment with topiramate activated the testicular pro-autophagy AMPK/mTOR pathway that was inhibited by cadmium administration. This was manifested by increased p-AMPK (Ser487)/total AMPK ratio [H (3, 20) = 5.71, *p* = 0.0054] (**A**) and lowered p-mTOR (Ser2448)/total mTOR ratio [H (3, 20) = 18.83, *p* < 0.0001] (**B**). Results are demonstrated as the mean ± standard error of the mean (SEM) for six rats in each experimental group. * *p* < 0.05, ** *p* < 0.01, **** *p* < 0.0001 signifies statistical significance versus the control group; ^#^ *p* < 0.05, ^##^ *p* < 0.01 signifies statistical significance versus cadmium chloride-treated group. AMPK, AMP-activated protein kinase; mTOR, mammalian target of rapamycin. TPM, topiramate; Cd, cadmium chloride.

The pro-autophagy AMPK/mTOR pathway [14,15] was examined for further characterization of the testicular autophagy events. In cadmium-intoxicated rats, testicular AMPK/mTOR pathway was inhibited compared to control rats. This was seen by a significant decline of p-AMPK (Ser487)/total AMPK ratio (by 44.7%, *p* < 0.01, Figure 8A) together with a significant increase of p-mTOR (Ser2448)/total mTOR ratio (by 184.3%, *p* < 0.0001, Figure 8B). These changes were counteracted by topiramate where AMPK/mTOR pathway was activated as demonstrated by a significant increase in p-AMPK (Ser487)/total AMPK ratio (by 70.5%, *p* < 0.05) together with a significant decrease in p-mTOR (Ser2448)/total mTOR ratio (by 35.3%, *p* < 0.01). These findings indicate that stimulation of AMPK/mTOR pathway by topiramate is, at least partly, involved in the observed increase of the pro-autophagy Beclin 1. At the cellular level, stimulation of pro-autophagy events plays a role in cleaning up the damage triggered by cadmium in rats.

## 3. Discussion

**Figure 9:**
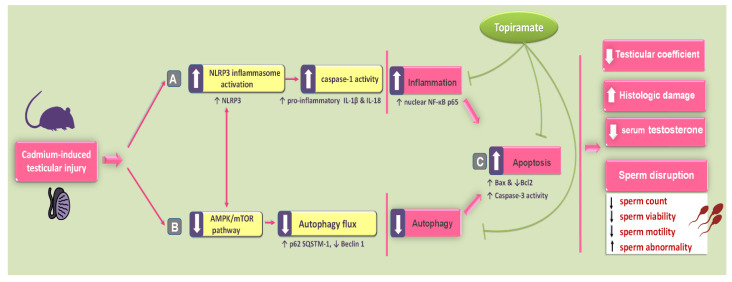
A summary of the molecular mechanisms that support the ameliorative impact of topiramate against cadmium-triggered testicular damage in rats. The observed improvement of testicular impairment and disrupted spermatogenesis were mediated by (**A**) Suppression of the NLRP3/caspase-1/IL-1β axis and curbing of NF-κB activation. (**B**) Inhibition of the pro-apoptotic events by lowering Bax protein expression and caspase-3 activity alongside upregulating the anti-apoptotic Bcl-2. (**C**) Stimulation of AMPK/mTOR-driven autophagy in the testicular tissues of rats. Notably, the observed autophagy stimulation is likely engaged in the suppression of NLRP3 inflammasome and associated pro-inflammatory events [7,15] and attenuation of the pro-apoptotic responses [13,15,21] in the testicular tissues of animals. Solid arrows demonstrate activation while blunt arrows depict inhibition.

The current work provides evidence that topiramate attenuates cadmium-triggered testicular injury in vivo in rats by combating inflammation and apoptotic cell death while activating the autophagy events. In perspective, these beneficial effects were interceded by the stimulation of autophagy-associated AMPK/mTOR pathway alongside the suppression of NLRP3/caspase-1/IL-1β-driven inflammation (Figure 9).

Three components of the NLRP3 inflammasome have been identified, including the ROS sensor NLRP3, the adapter subunit ASC, and the effector caspase-1. In fact, activated NLRP3 inflammasome is switched on by two steps: first, by a “priming” stimulus, including cellular stress stimuli such as ROS and danger-associated molecular patterns (DAMP) [6,15]. This step is required to induce the protein expression of the three critical inflammasome components. In this context, DAMP binds to the toll-like receptor-4 ensuing activation of the NF-κB pathway which culminates in enhanced transcription of NLRP3, ASC, and pro-caspase-1 [6,7]. This is followed by a subsequent “activating” stimulus that causes inflammasome assembly into one NLRP3 inflammasome complex. The later event instigates the proteolytic cleavage of pro-IL-1β and pro-IL-18 into IL-1β and IL-18, respectively, provoking severe inflammatory responses and cell pyroptosis—an inflammatory form of cell death—in the testicular tissue [15]. Coinciding with these data, the present work demonstrated that cadmium triggered testicular NLRP3 activation with concomitant caspase-1 activation and IL-1β/IL18 production. These deleterious events were curtailed by topiramate administration. In fact, the marked anti-inflammatory actions of topiramate and associated suppression of pro-inflammatory cytokines have been demonstrated as an effective tool for the mitigation of abdominal aortic aneurysm [25] via favoring the conversion of the pro-inflammatory M1 to the anti-inflammatory M2 macrophage phenotype. In fact, the GABA_A_-receptor agonist feature of topiramate has been reported to mediate its anti-inflammatory and immune-modulating actions against cardiac post-infarction inflammation. This has been evidenced by the expression of the anti-inflammatory GABA_A_ receptor in monocytes/macrophages [24]. Likewise, dampening the pro-inflammatory Jannus kinase (JNK) and NF-κB pathways by topiramate has interceded its favorable effects in skin wound healing [26] and ethanol-triggered gastric injury [30], respectively. Notably, the observed NLRP3 inflammasome inhibition by topiramate can activate the autophagy flux, culminating in attenuation of cadmium-induced testicular dysfunction and impaired spermatogenesis. Indeed, the interplay between the NLRP3 inflammasome and autophagy has been characterized. In perspective, the interventions that can activate testicular autophagy flux have been reported to limit NLRP3 inflammasome activity and downregulate pyroptosis [7]. 

In response to cadmium, excessive mitochondrial apoptotic events have been characterized [4,8,9,14]. Evidence exists that excessive pro-apoptotic events in germ and Leydig cells have been associated with impaired spermatogenesis [9,20,31]. In this regard, overexpression of the pro-apoptotic Bax and downregulation of Bcl-2 expression have been reported to disrupt mitochondrial membrane integrity, resulting in cytochrome C release and activation of the executioner caspase-3 activity [14]. Consistently, the current data revealed activation of mitochondrial apoptosis in testicular tissues in vivo; events that were attenuated by topiramate administration. The present data also pointed out that topiramate opposed the pro-apoptotic responses triggered by cadmium as evidenced by upregulated expression of the anti-apoptotic signal Bcl-2 alongside downregulating Bax and dampening caspase-3 activity. Consistently, the anti-apoptotic actions of topiramate advocated its beneficial effects in several pathological models in rodents. These included experimental models of ethanol-evoked gastric injury [30], infrarenal aortic occlusion-triggered intestinal damage [32], post-operative cognitive dysfunction [33], amyloid beta 42-induced Alzheimer’s disease [28], Huntington’s disease-like neuropathology [27], and methylphenidate-induced hippocampal damage [29]. The observed activation of testicular autophagy by topiramate can favor the dampening of apoptosis. This can be perceived by the fact that autophagy stimulation has been reported to antagonize apoptosis and favors survival of germ cells, Leydig cells [34], and mouse spermatocytes [35]. In Sertoli cells, activation of autophagy has been characterized to dampen cadmium-triggered apoptosis [8]. 

The classical purpose of autophagy is maintaining cellular homeostasis when cells are subjected to stressors by eliminating damaged mitochondria, the main source of ROS alongside damaged/misfolded proteins [11,12,19]. These autophagy events rescue cells from cell death, thereby serving as pro-survival signals that suppress apoptotic cell death [15,34]. On the other hand, excessive autophagy has been envisioned as a newly characterized mechanism for provoking cell death, thereby offering an alternative cell-death pathway [34]. In rodent models of cadmium-evoked testicular injury, the literature revealed impaired [13,20] as well as overactive autophagy [34,36]. Thus, further elucidation of the role of autophagy in the pathogenesis of cadmium-induced testicular damage was needed. The present data elucidated defective testicular autophagy in cadmium-intoxicated animals. This was manifested by accumulation of the negative autophagy marker p62 SQSTM1. In fact, several studies have demonstrated that impaired autophagy flux is linked to lowered testosterone levels and defective spermatogenesis [13,20]. The cadmium-triggered autophagy impairment has been mediated by disrupted calcium-dependent lysosome fusion with autophagosome [17] and excessive lysosomal membrane damage [18]. As a result of impaired autophagy flux, accumulation of damaged mitochondria and ROS have been reported to provoke the signaling of apoptosis [15]. The later response triggers activation of the executioner caspase-3 that degrades Beclin 1, thereby abrogating the initiation of autophagy events [37]. 

Several studies have revealed that therapeutic interventions that stimulate autophagy flux in germ cells have been proven effective tools for improving cadmium-induced testicular impairment [13,20,21]. Conceptually, autophagy activation triggers the elimination of damaged mitochondria, the principal source of ROS generation, thereby suppressing the apoptotic machinery [13,15,21]. Moreover, the autophagy process provides a tool to enhance cellular energy generation by recycling/degrading misfolded proteins through the autophagosome/lysosome axis [11,12]. Coinciding with these data, the present work revealed activation of testicular autophagy by topiramate administration as proven with upregulated Beclin 1 expression and decreased accumulation of p62 SQSTM1. These observations are in line with the pro-autophagic features of topiramate that were demonstrated in APPswe/PS1dE9 transgenic model of Alzheimer’s disease in vivo and SH-SY5Y cells in vitro as evidenced by upregulating the positive autophagy marker Beclin 1 [38]. In fact, the role of Beclin 1 as a positive autophagy marker has been characterized in the autophagy process at the stage of autophagosome sequestration [14]. Meanwhile, the role of the adaptor signal p62 SQSTM1 in the detection of ubiquitin-tagged proteins has been defined to aid in their elimination. During this process, the p62 SQSTM1 itself is degraded, thereby serving as a negative autophagy marker [11,14]. Notably, the observed topiramate-evoked testicular autophagy activation has been linked to increased steroidogenesis and testosterone production [15]. In this context, autophagy stimulation has been reported to augment cholesterol-trafficking and its delivery to Leydig cells and associated steroidogenesis enhancement [39]. This takes place by the effective interaction of autophagosome-mediated cholesterol trafficking and the lysosomal system [39,40]. 

The crosstalk between autophagy and apoptosis has been reported to be tightly controlled [14,19]. The relationship between autophagy and apoptosis processes may overlap [41]. When cells are subjected to mild stress, autophagy events prevail to rid the cells of stress signals. Thus, under this condition, autophagy serves as a cell-survival pathway likely via the removal of damaged fragments, thereby, switching off apoptosis [11,19]. However, when cells are exposed to overwhelming stress signals and cells are no longer able to defend themselves against cellular damage, activation of apoptosis ensues or even activation of both overactive autophagy and apoptosis prevails, culminating in cell death [42]. Notably, the interplay between autophagy and apoptosis has been demonstrated in several testicular disorders [13,20]. Consistent with the present mitigation of cadmium-induced testicular dysfunction by topiramate thanks to its pro-autophagic and anti-apoptotic actions, previous literature revealed that effective agents can activate autophagy and dampen apoptotic cell death in testicular dysfunction models [21]. Among the key factors connecting autophagy and apoptosis, Beclin 1/Bcl-2 complex serves as a crucial factor [41]. Evidence exists that the interaction between Beclin 1 and Bcl-2 prevents the pre-autophagosome structure assembly, thereby blocking autophagy flux [41]. Contrarily, phosphorylation of Bcl-2, by Janus kinase 1 (JNK1), dampens the affinity of Bcl-2 to Beclin 1, liberating the later signal for autophagy activation [43]. Evidence also exists that Klotho, an antiaging signal, triggers autophagy activating by disrupting Beclin 1/Bcl-2 interaction resulting in favorable tissue protection in rodents [44]. 

The present work further explored the potential involvement of AMPK/mTOR pathway in the observed activation of autophagy in the testicular tissues of rats. Indeed, evidence has characterized AMPK/mTOR as an essential pro-autophagic pathway in several testicular pathology models [7,14,15], including cadmium-induced testicular damage [20]. Consistently, the current data demonstrated that cadmium triggered testicular suppression of AMPK/mTOR pathway; an event that was counteracted by topiramate administration. This later event was evidenced by increased phosphorylation of AMPK alongside lowered phosphorylation of mTOR, thereby promoting autophagy flux activation. By serving as a cellular energy sensor, AMPK is a positive regulator of autophagy to boost energy production by recycling damaged proteins [15,45]. This takes place by lowering the phosphorylation of the negative autophagy regulator mTOR [45,46]. Interestingly, activation of AMPK/mTOR and associated autophagy stimulation has been reported to ameliorate Leydig cell damage in vitro [15] and cadmium-induced testicular injury in vivo [20]. Notably, the crosstalk between autophagy activation and pyroptosis suppression in Leydig cells has been demonstrated via ROS/AMPK/mTOR axis, culminating in the restoration of steroidogenesis and testosterone production [15]. Notably, the observed activation of AMPK/mTOR pathway has been previously described for topiramate in brain tissue of APPswe/PS1dE9 transgenic model of Alzheimer’s disease as evidenced by increased phosphorylation of AMPKα [38].

## 4. Materials and Methods

### 4.1. Chemicals

Janssen-Cilag Pharmaceuticals (Raritan, NJ, USA) provided topiramate while Sigma-Aldrich (St. Louis, MO, USA) procured cadmium chloride (CdCl_2_; Cat. # 202908). Other chemicals were provided as the highest quality available. 

### 4.2. Animals

Animal procedures and care were carried out according to institutional guidelines that complied with the Guide for the Care and Use of Laboratory Animals, United States National Institutes of Health (Publication No. 85-23, revised 1996). The Research Ethics Committee at Taif University authorized the present study protocol (Approval ID number 43-559).

The present work included 24 male Wistar rats weighing 170–210 g. During the entire experimental protocol, animals were housed at constant temperature (21 ± 2 °C), relative humidity (55%), and 12 h day/12 h night cycle. The animals were given free access to food and drinking water after a week of acclimatization.

### 4.3. Experimental Design and Protocol

Random allocation of experimental animals was carried out by a blinded technician to the study design. As depicted in Table 1.

After the experimental period, fasting of animals was performed overnight and body weight was listed. Following thiopental sodium anesthesia (50 mg/kg; i.p.) [49], blood was taken through the retro-orbital route for serum separation, and euthanization was then applied by cervical dislocation. Following laparotomy, surgical removal of testes was carried out and the testicular coefficient was calculated (weight of the 2 testes (g)/total body weight (kg)) [50]. The semen analysis was performed after opening/evacuation of the cauda epididymis. For histopathology and immunohistochemistry, preservation of the left testis in 10% neutral buffered formalin was performed from 3 randomly chosen rats per group. Meanwhile, the biochemical determinations were performed in the homogenates of right testis. In perspective, homogenization of part of the right testis was applied in lysis buffer (10 mM Tris, pH 7.4) complemented with protease inhibitors for ELISA assays. Meanwhile, homogenization of another part of the right testis was performed in potassium phosphate buffer (50 mM, pH 7.4) for the remaining assays. Determination of testosterone was carried out in serum.

### 4.4. Evaluation of Semen Parameters

The sperm parameters, including sperm count, viability, motility, and abnormality were determined as previously characterized [51]. The cauda epididymis was opened and counting of sperms was carried out by microscopy using the 40 × lens. Sperm viability in terms of % vital sperms was determined in Eosin-Negrosin stain where dead sperms were stained with the later dye. Evaluation of motility of sperms was performed within 4 min of their isolation, by mixing one drop of 2.9% sodium citrate solution with one drop of seminal fluid on a 37 °C preheated slide. Meanwhile, the percentage of abnormal sperms was recorded.

### 4.5. Serum Testosterone Determination

Determination of serum testosterone was carried out using Cusabio ELISA kit (Catalog # CSB-E05100r, Cusabio Technology, Houston, TX, USA) as instructed by the supplier.

### 4.6. Estimation of Inflammatory Markers

Estimation of testicular interleukin 18 (IL-18) and interleukin 1 beta (IL-1β) was carried out using ELISA kits procured from Cusabio Technology (Catalog # CSB-E04610 and Catalog # CSB-E04610r, respectively, Cusabio Technology, Houston, TX, USA). Optical density determination of the final color was applied at 450 nm. Determination of the nuclear factor kappa B p65 (NF-κBp65) was performed in the nuclear compartment. To this end, extraction of the nuclear proteins was applied by the Cayman nuclear extraction kit (Catalog # 10009277, Cayman Chemical Company, Ann Arbor, MA, USA). Then, quantification of NF-κBp65 protein expression was applied using Elabscience ELISA kit (Catalog # E-EL-R0674, Elabscience ELISA kit, Wuhan, China) as guided by the provider. Optical density determination of the final color was performed at 450 nm.

### 4.7. Determination of the Activity of Caspase-1 and Caspase-3

Estimation of testicular caspase-1 activity was carried out by a colorimetric kit procured from R&D Systems (Catalog # K111-100, R&D Systems, Minneapolis, MN, USA). Optical density determination of the final color was applied at 405 nm. Measurement of testicular caspase-3 activity, a well-characterized pro-apoptotic signal, was established with Sigma-Aldrich colorimetric assay kit as instructed by the provider (Catalog # CASP-3-C, Sigma-Aldrich, St. Louis, MO, USA). Optical density determination of the final color was applied at 405 nm. For both parameters, the values were expressed as fold-change as guided by the manufacturer’s instructions.

### 4.8. Estimation of Autophagy Markers 

The protein expression of p-mTOR (Ser2448) and total mTOR was measured using specific ELISA kits (Catalog # 7976C and Catalog # 7974C, respectively, Cell Signaling Technology, Danvers, MA, USA). Likewise, the protein expression of p-AMPK(Ser487) and total AMPK was quantified using RayBiotech ELISA kit (Catalog # PEL-AMPKA-S487-T, RayBiotech., Peachtree Corners, GA, USA). For these kits, optical density determination of the final color was performed at 450 nm. The quantification of the protein expression of p62 SQSTM1 (Catalog # MBS3809397, MyBioSource, San Diego, CA, USA) and Beclin1 (Catalog # EL002658RA, Cusabio Technology, Houston, TX, USA) was performed with the corresponding ELISA kit, according to the provider’s instructions. Optical density determination of the final color was performed at 450 nm. 

### 4.9. Estimation of Cadmium Metal Content in Testis

The content of cadmium metal was measured in the testicular tissue as described [52]. In brief, 1 M Nitric acid was added for the digestion of part of the testicular tissue. Then, the procedures included ashing at 150 °C, and metal detection at 228.8 nm (PerkinElmer 3100 Atomic Absorption Spectrophotometer, Akron, OH, USA). 

### 4.10. Histopathology

After tissue fixation in 10% formol saline, the testicular specimens were dehydrated in alcohol, xylene cleared, and paraffin-embedded [53,54]. Then, 5 µm sections were prepared, deparaffinized, and stained with hematoxylin and eosin (H&E). The sections were examined by light microscopy (Leica Microsystems GmbH, Wetzlar, Germany) and the images were captured by a digital camera. To avoid bias, image analysis was applied in a blinded manner.

### 4.11. Immunohistochemistry

The testicular protein expression of the nucleotide-binding domain (NOD)-like receptor family, pyrin domain-containing 3 (NLRP3), B-cell lymphoma-2 protein (Bcl-2), and Bcl-2 associated x protein (Bax) was assessed by immunohistochemistry as described [55,56]. To this end, testicular cross-sections from all experimental groups were deparaffinized, rehydrated, and treated with 3% hydrogen peroxide solution for 20 min to block the endogenous peroxidase activity. Following antigen retrieval with citrate buffer (pH 6.0) in the microwave, the sections were blocked with 5% bovine serum albumin. Anti-NLRP3 (Catalog # GTX00763, GeneTex Inc., Irvine, CA, USA, 1:100 dilution), anti-Bcl-2 (Catalog # PA1-30411, 1:100 dilution, Thermo Fisher Scientific, Waltham, MA, USA) or anti-Bax (Cat. # MA5-14003, 1:100 dilution, Thermo Fisher Scientific, Waltham, MA, USA) were applied in humidified chambers against corresponding rat antigens. This was followed by washing and treatment with HRP-tagged secondary antibody for 30 min. Staining of target signals was carried out with diaminobenzidine (DAB) for 10 min and counterstaining was done with hematoxylin. Finally, the sections were examined by Leica light microscope fitted with a full-HD microscopic imaging system for image capturing (Leica Microsystems GmbH, Wetzlar, Germany). At 400× magnification, six non-overlapping fields were photographed per specimen. To ensure specificity of the utilized antibodies, a negative control was also established using normal rabbit serum instead of the primary antibodies. Data analysis was carried out by a blinded observer to avoid bias using the Leica Application Module to quantify the immunostaining of target proteins. 

### 4.12. Statistics

The results were displayed as mean ± standard error of the mean (SEM). To check the normal distribution of the findings, Shapiro–Wilk test was applied. Statistical analysis was applied by one-way analysis of variance followed by Tukey’s test. The minimum acceptable threshold of statistical significance was established at *p* < 0.05.

## 5. Conclusions

The present work demonstrates the ameliorative impact of topiramate against cadmium-evoked testicular injury and impaired spermatogenesis in vivo. To put this into perspective, topiramate exerted anti-inflammatory, anti-apoptotic, and pro-autophagic actions that mediated its favorable effects against the testicular insult. Notably, inhibition of NLRP3/caspase1/IL-1β axis alongside activation of the pro-autophagic AMPK/mTOR pathway contributed to the observed beneficial effects of topiramate. Hence, topiramate may serve as a promising adjunct tool against testicular injury, particularly, in patients with co-existing neurological disorders. Future studies are needed to characterize the effects of topiramate against cadmium-evoked testicular toxicity at multiple doses. In fact, the present work is a proof-of-concept approach and supplemental studies are warranted to define the exact molecular pathways involved in topiramate’s actions including autophagy events such as LC3I/II and LAMP protein expression alongside the impact of topiramate on Beclin 1/Bcl-2 protein–protein interaction. 

## Figures and Tables

**Table 1:** The Experimental design.

Group	N	Description
Control group	6	The oral vehicle of topiramate was received by gavage (0.5% carboxymethyl cellulose; CMC; 10 mL/kg/day). Meanwhile, normal saline vehicle (10 mL/kg/day) was given by gavage. The treatments lasted for 2 months.
Control + TPM group	6	Topiramate suspended in CMC was received by gavage (50 mg/kg/day, 10 mL/kg). Meanwhile, normal saline (10 mL/kg/day) was given by gavage. The treatments lasted for 2 months.
Cd group	6	0.5% CMC (10 mL/kg/day) was received by gavage. Meanwhile, a gavage of cadmium chloride solution (5 mg/kg/day; 10 mL/kg) was given daily. The treatments lasted for 2 months. The dose of cadmium chloride is compliant with previous studies [20,47].
Cd + TPM group	6	Topiramate (50 mg/kg/day, 10 mL/kg) was received by gavage. Meanwhile, a gavage of cadmium chloride solution (5 mg/kg/day; 10 mL/kg) was given daily. The treatments lasted for 2 months. The dose of topiramate was based on the previously reported efficacy against several pathological models [27,29,48].

## Data Availability

Data are contained within the article.
